# The protein gap—increasing protein intake in the diet of community-dwelling older adults: a simulation study

**DOI:** 10.1017/S1368980021004134

**Published:** 2022-02

**Authors:** Marije H Verwijs, Marian AE de van der Schueren, Marga C Ocké, Jacco Ditewig, Joost O Linschooten, Annet JC Roodenburg, Lisette CPGM de Groot

**Affiliations:** 1 HAN University of Applied Sciences, Nijmegen, Netherlands; 2 Wageningen University and Research, Stippeneng 4, 6708WE Wageningen, Netherlands; 3 National Institute for Public Health and the Environment, Bilthoven, Netherlands; 4 HAS University of Applied Sciences, ‘S-Hertogenbosch, Netherlands

**Keywords:** Protein intake, Older adults, Simulation model, Food consumption survey

## Abstract

**Objective:**

Approximately 50 % of Dutch community-dwelling older adults does not meet protein recommendations. This study assesses the effect of replacing low protein foods with protein-rich alternatives on the protein intake of Dutch community-dwelling older adults.

**Design:**

The Dutch National Food Consumption Survey—Older Adults 2010–2012 (DNFCS-OA) was used for scenario modelling. Dietary intake was estimated based on two 24-h recalls. Commonly consumed products were replaced by comparable products rich in protein (scenario 1), foods enriched in protein (scenario 2) and a combination of both (scenario 3). Replacement scenarios were confined to participants whose dietary protein intake was < 1·0 g/kg BW/d (*n* 391). Habitual protein intake of all older adults was estimated, adjusting for effects of within-person variation in the 2-d intake data.

**Setting:**

A simulation study based on the DNFCS-OA.

**Participants:**

727 Dutch community-dwelling older adults aged 70+.

**Results:**

Mean protein intake of the total population increased from 1·0 to 1·2 g/kg BW/d (scenarios 1 and 2) and to 1·3 g/kg BW/d (scenario 3). The percentage of participants with intakes of ≥ 1·0 g/kg BW/d increased from 47·1 % to 91·4 %, 90·2 % and 94·6 %, respectively, in scenarios 1, 2 and 3. The largest increases in protein intake were due to replacements in food groups: yoghurt, cream desserts and pudding, potatoes, vegetables and legumes and non-alcoholic beverages and milk in scenario 1 and bread; yoghurt, cream desserts and pudding and soups in scenario 2.

**Conclusions:**

This simulation model shows that replacing low protein foods with comparable alternatives rich in protein can increase the protein intake of Dutch community-dwelling older adults considerably. Results can be used as a basis for nutritional counselling.

Vitality and independence are important determinants of healthy ageing. However, the ageing process is naturally associated with loss of muscle mass, reduced strength and physical endurance^(1)^. A healthy diet in combination with physical exercise can limit age-related muscle decline and help retain optimal muscle function, with a key role for dietary protein^(2,3)^. The current WHO recommendation for protein is 0·8 g/kg body weight/d (g/kg BW/d), for both men and women^(4)^. Expert groups recommend a higher intake of 1·0–1·2 g/kg BW/d for healthy older adults, and even 1·2–1·5 g/kg BW/d for older adults who are malnourished or at risk of malnutrition^(5,6)^.

The Dutch National Food Consumption Survey Older Adults (2010–2012; DNFCS-OA) revealed that 15·4 % of older adults did not meet the recommended daily intake of 0·8 g/kg BW/d^(7)^. Furthermore, approximately 50 % of the participants of the DNFCS-OA did not meet the higher protein recommendation of ≥ 1·0 g/kg BW/d^(7)^. In accordance, the risk of protein–energy malnutrition remains high among community-dwelling older adults: varying from 7 to 12 % among Dutch community-dwelling older adults without home care to 30–40 % among older adults receiving home care^(8)^. Protein–energy malnutrition may result in reduced functioning of the immune system, impaired muscle function, fatigue, impaired wound healing and depression^(9–11)^. Protein–energy malnutrition, therefore, influences the physical, mental and social well-being of older adults and can pose a threat to independence and quality of life^(11,12)^. This emphasises the importance of an adequate intake of dietary protein. Despite the increasing availability of protein-rich and protein-enriched food products, it is still unclear to what extent such products could help to improve protein intakes if chosen instead of regularly consumed products lower in protein. A study by Beelen *et al.* (2017) revealed that older adults often experience difficulties in adopting and applying changes in their diets, which suggests that staying close to their existing dietary pattern and only making subtle changes, may be most effective in increasing their protein intake^(13)^. Accordingly, this study aims to assess the potential effect of replacement of commonly consumed foods low in protein content by comparable products high in protein content and/or protein-enriched products on daily protein intake of Dutch community-dwelling older adults.

## Methods

### Study population

The sampling frame for DNFCS-OA consisted of the population registers of fifteen municipalities in the Netherlands, covering five regions and three region-specific classes of address density. Within each municipality, a sex and age-stratified sample was randomly drawn from the population register. This was the sampling base from which a market research organisation recruited participants. At first, potential participants received an invitation with information leaflet by mail with an enclosed reply letter. Because response was very low, recruitment changed to face-to-face visiting after sending written information about the study. The DNFCS-OA included 739 community-dwelling older adults living in the Netherlands, and the overall response rate was 25·9 %. Mean age was 77·1 (sd = 5·2 years) and 41·9 % were male. The survey was a nationwide cross-sectional study, representative for region, address density and age. Height and weight were measured during a home visit and mean BMI was 27·4 kg/m^2^ (sd = 3·8). The population consisted of 97·0 % Dutch participants. Over 50 % of the population was married or lived together. Of all participants, 52·9 % had a mean protein intake below 1·0 g/kg BW/d (*n* 391). Almost all participants (96·6 %) scored high on the Mini-Mental State Examination score, indicating a low risk of dementia. Furthermore, 95·7 % of the participants reported not to have eating and drinking difficulties. Other characteristics and specific details on the recruitment of the participants are described elsewhere^(7)^.

### Dietary intake

Data on dietary intake were collected through two non-consecutive dietary 24 h-recalls performed by trained dietitians. Recalls were spread equally over all days of the week and throughout the year and were assisted by food diaries. Participants were asked to record and memorise all foods consumed from the moment of getting up on 1 d to the moment of getting up the next day. During the 24-h recalls, participants were asked about the time and place of food consumption occasions. Furthermore, they were asked to describe the type of food consumed using descriptors to specify the foods and to give an indication of the quantity of the food consumed through household measures, units, by weight/volume or by photo series showing a range of quantities. Detailed information on data collection of the DNFCS-OA can be found elsewhere^(7)^. Dietitians entered the recall data directly into the computer and products were grouped using EPIC-Soft (IARC©) food group classification^(14)^. All foods were linked to 1347 food items from the extended version of Dutch Food Composition Database of 2011 (The National Institute for Public Health and the Environment, 2011). Accordingly, total energy, macro- and micronutrient intakes were calculated.

### Data analysis

Participants were excluded in the current data analysis when BW was unknown (*n* 12). For each of the remaining 727 participants, protein intake/kg BW/d was calculated for each 24-h dietary recall and averaged over the two recalls. Food replacements in all three scenarios were restricted to participants with an average protein intake < 1·0 g/kg BW/d (*n* 391). Participants with a protein intake of ≥ 1·0 g/kg BW/d were not included in the scenarios. For each of three scenarios, every food item within a specific food group with a protein content below the 75th percentile of the protein contents in that food group was replaced by a high protein alternative product from the same food group. Food groups were included in the different scenarios based on the results of the DNFCS-OA^(7)^ and a study by Hung *et al.* ^(15)^ and their role in the eating pattern in the Netherlands. First a long list of potential food groups, based on either the food groupings in EPIC-Soft or in NEVO, was prepared. Food groups were included when intakes of that food group contributed at least 10 % to total food consumption during a specific meal moment^(7)^, or when they contributed most to total intake in grams of a specific meal moment^(15)^. Only food groups that contributed much to protein intake were included in the scenarios since food groups low in protein content (e.g. fruit or fats) or food products from food groups consumed in small quantities (e.g. herbs) would not have a large impact on protein intake.

Products were substituted on the basis of weight (g) consumed. The first scenario focussed on replacement by high protein foods, the second scenario on protein-enriched foods and the third scenario on a combination of both.

#### Scenario 1

In scenario 1, replacements were made in nine different food groups. The alternative food product has a protein content > P75 of protein content in that food group. To design a feasible scenario in daily practice, the alternative food product should be familiar to the target group and therefore alternative food products were commonly consumed food products within the DNFCS-OA. Food groups and their alternative foods of scenario 1 are presented in Table 1.

**Table 1:** Scenario 1: food groups to be replaced and their protein-rich alternatives

Food group	Group classification*	Protein P75 (g/100 g)	Scenario 1 protein-rich alternatives	Energy (kcal/100 g)	Protein (g/100 g)
Bread	EPIC-Soft	11·0	Bread multigrain, with seeds	261	12·3
Breakfast cereals	EPIC-Soft	11·0	Oatmeal	373	12·8
Cakes + chocolate + candy bars	EPIC-Soft	6·9	Muesli bar with chocolate	452	7·7
Dairy: yoghurt + cream desserts and pudding	EPIC-Soft	4·0	Quark, low fat	58	8·5
Non-alcoholic beverages + dairy: milk†	EPIC-So0ft	0·9	Yoghurt drink with sweetener (Optimel, FrieslandCampina)	30	3·1
Nuts and seeds + savoury snacks	NEVO	18·5	Peanuts, unsalted	627	25·2
Potatoes, vegetables and legumes†	EPIC-Soft	3·0	Lentils	99	8·8
Soups	EPIC-Soft	4·2	Soup with meat, vegetables and noodels	42	4·4
Spreads: cheese + savoury spreads + sweet spreads†	NEVO	25·5	Cheese, 30+	289	30·4

* Group classification: based on the products that were covered, groups were chosen from NEVO of EPIC-Soft classification.

† Included in scenario 3.

#### Scenario 2

In scenario 2, replacements were made in six different food groups, based on EPIC-Soft classification. The alternative foods have a protein content > P75 in that food group and foods had to be familiar to older adults, that is, be similar to the foods they replace. Food groups and their alternative foods of scenario 2 are presented in Table 2.

**Table 2:** Scenario 2: food groups to be replaced and their protein-rich alternatives

Food group	Group classification*	P75 (g/100 g)	Scenario 2 protein-enriched alternatives	Energy (kcal/100 g)	Protein (g/100 g)
Bread†	EPIC-Soft	11·0	White Bread (Carezzo)	255	27·0
Cakes + chocolate + candy bars	EPIC-Soft	6·9	‘Bouwsteentje’ (De Bakker BV)	352	15·0
Ice cream	EPIC-Soft	4·3	Ice cream, vanilla (Carezzo)	176	11·5
Non-alcoholic beverages	EPIC-Soft	0·4	Apple juice (Carezzo)	79	6·7
Soups†	EPIC-Soft	4·2	Funghi soup (Carezzo)	66	6·9
Yoghurt + cream desserts and pudding†	EPIC-Soft	4·0	BonDuo Breakfast	133	12·0

* Group classification: based on the products that were covered, groups were chosen from NEVO of EPIC-Soft classification.

† Included in scenario 3.

#### Scenario 3

Replacements were made in six different food groups in scenario 3. Choice of food groups and alternative foods was based on the top 3 food groups of scenarios 1 and 2 for which replacements resulted in the largest increase in protein intake. The top 3 of scenario 1 included the food groups *Yoghurt, cream desserts and pudding*, *Potatoes, vegetables and legumes* and *Non-alcoholic beverages and milk*. However, since the food group *Yoghurt, cream desserts and pudding* was in the top 3 in both scenarios, *Savoury spreads, sweet spreads and cheese* was the fourth most contributing food group in scenario 1 and was chosen as an alternative. Besides *Yoghurt, cream desserts and pudding*, the top 3 of scenario 2 included the food groups *Soups* and *Bread*. Food groups and alternative foods included in scenario 3 are presented in Tables 1 and 2, indicated with an †-sign.

For each scenario, protein intake for each consumption day was determined per particpant. For the whole population of older adults and in all three scenarios, the distribution of the habitual protein intake adjusted for within-person variability was estimated using SPADE software^(16)^, a statistical program to estimate habitual dietary intake. The proportion of older adults with a habitual protein intake below a cut-off value of 1·0 g/kg BW/d was estimated and compared to the original scenario. Also, the impact of the exchange of protein sources on mean intakes of other macro- and micronutrients was assessed. Here, average intake over 2 d was used to calculate the mean intakes. Results were weighed for socio-demographic deviances and deviances in the day of the week and season. Besides the SPADE software, data were analysed using SAS Software (SAS version 9.4, SAS Institute).

## Results

### Simulation model

The number of replacements in the three different scenarios increased from 2686 in scenario 2, to 4135 in scenario 3 and 5535 in scenario 1 (Table 3).

**Table 3:** Food groups, alternative foods and frequencies of replacement

Food group	Scenario 1			Scenario 2			Scenario 3	
	Total replacements (*n*)	Replacements per person*/d (mean *n*)	Food group	Total replacements (*n*)	Replacements per person*/d (mean *n*)	Food group	Total replacements (*n*)	Replacements per person*/d (mean *n*)
Bread	850	2·2	Bread	850	2·2	Bread	850	2·2
Breakfast cereals	67	0·2	Cakes	883	2·3	Non-alcoholic beverages + dairy: milk	582	1·5
Cakes + candy bars + chocolate	1150	2·9	Ice cream	29	0·1	Potatoes + vegetables + legumes	1429	3·7
Non-alcoholic beverages + dairy: milk	582	1·5	Non-alcoholic beverages	302	0·8	Soups	210	0·5
Nuts and seeds + savoury snacks	183	0·5	Soups	210	0·5	Spreads: sweet + savoury spreads + cheese	652	1·7
Potatoes + vegetables + legumes	1429	3·7	Yoghurt + cream desserts and pudding	412	1·1	Yoghurt + cream desserts and pudding	412	1·1
Soups	210	0·5						
Spreads: sweet + savoury spreads + cheese	652	1·7						
Yoghurt + cream desserts and pudding	412	1·1						
Total	5535	14·2	Total	2686	6·9	Total	4135	10·6

* Number of replacements in respondents with an average intake below 1·0 g/kg BW/d (*n* 391).

#### Effects on mean protein intake

##### Scenario 1: protein-rich foods

Replacing foods from nine different food groups in participants with a protein intake < 1·0 g/kg BW/d (*n* 391) with protein-rich alternatives resulted in an increase in mean habitual protein intake in the total population (*n* 727) from 1·0 to 1·2 g/kg BW/d or 76·5 to 89·7 g/d. Overall, the percentage of participants who met the recommended intake of 1·0 g/kg BW/d increased from 41·1 % to 91·4 %. Replacements in food groups contributing most to the increase in protein intake were *Yoghurt, cream desserts and pudding*; *Potatoes, vegetables and legumes* and *non-alcoholic beverages and milk*.

##### Scenario 2: protein-enriched foods

In scenario 2, consumed foods in six food groups were replaced with protein-enriched products, resulting in an increase in mean habitual protein intake from 1·0 to 1·2 g/kg BW/d and from 76·5 to 91·8 g/d. In this scenario, 90·2 % of the participants reached a mean habitual protein intake ≥ 1·0 g/kg BW/d. The three food groups with the highest impact on increasing protein intake were *bread*; *yoghurt, cream desserts and pudding* and *soups*.

##### Scenario 3: top 3’s of scenarios 1 and 2

Scenario 3 included the top three contributing food groups of scenario 2, and three other most contributing food groups of scenario 1. This scenario resulted in a mean habitual protein intake of 1·3 g/kg BW/d and 93·9 g/d with 94·6 % of the participants achieving a protein intake ≥ 1·0 g/kg BW/d.

### Intake of other macro and micronutrients

Evaluation of differences in intake of other macro and micronutrients was based on the average of the 2-d 24 h recalls. Mean energy intake (kJ (kcal) and sd) and mean intake of carbohydrates, total fat and dietary fibre was similar in all scenarios and amounted to approximately 8375 kJ (2000 kcal) sd = 1900, 45 En %, 35 En % and 20–24 g, respectively. Dietary fibre intake was low in all scenarios compared to the recommendation. Mean intakes of other macronutrients were in line with the recommended intake. Intakes of macronutrients are listed in Table 4. Most micronutrients remained comparable to the original scenario (Appendix I). Copper and iodine intakes increased substantially in scenario 1 compared to the original scenario, while iodine intake was much lower in scenarios 2 and 3. Mean selenium intake varied through all scenarios being lowest and lower than recommended in the original scenario and scenario 2. Calcium intake increased considerably in scenarios 1 and 3. In all scenarios, mean vitamin C intake was higher than the average requirement.

**Table 4:** Mean intake and sd of other macro and micronutrients based on 2 d intake per scenario (*n* 727)

Nutrient	Norm intakes^(31)^	Average intake of 2 d											
		Original scenario			Scenario 1			Scenario 2			Scenario 3		
		Mean		sd	Mean		sd	Mean		sd	Mean		sd
Energy in kJ (kcal)	Men: 2500 Women: 2000	8264	1974	1948	8545	2041	1932	8503	2031	1928	8587	2051	1878
Total protein (g)	46	76·5		19·5	90·7		18·3	92·6		21·7	95·6		21·4
Total fat (g)	20–35 En %	76·4	34·8 En %	24·2	78·6	34·7 En %	25·0	77·8	34·5 En %	23·7	76·7	33·7 En %	24·0
SFA (g)	As low as possible	29·6		10·8	29·3		10·8	30·1		10·6	29·6		10·6
Mono-unsaturated fatty acids (g)		25·0		8·9	26·7		10·1	23·8		9·2	24·2		9·2
Poly-unsaturated fatty acids (g)		15·0		6·9	15·9		6·9	14·5		7·0	14·6		7·0
Trans fatty acids (g)	As low as possible	1·4		0·8	1·2		0·7	1·2		0·7	1·3		0·8
Carbohydrates (g)	45–60 En %	213·1	43·2 En %	56·0	209·0	41·0 En %	55·6	207·4	40·8 En %	57·0	211·2	41·2 En %	55·3
Dietary fibre (g)	25	20·8		6·5	23·8		6·9	20·9		6·7	23·0		7·1

## Discussion

This study assessed the effects of replacing foods low in protein content by commonly consumed protein-rich and/or protein-enriched alternative foods on daily protein intake of Dutch community-dwelling older adults. Outcomes showed that replacing low protein foods within six to nine food groups with similar high protein alternatives for participants with a mean protein intake < 1·0 g/kg BW/d, the protein intake of Dutch community-dwelling older adults can be increased considerably. The proportion of older adults complying with a protein intake of at least 1·0 g/kg BW/d increased from 47·1 % to approximately 90 % in all three scenarios of the simulation model.

Although the three different scenarios showed similar results with regard to mean protein intake and proportion of participants with a mean intake ≥ 1·0 g/kg BW/d, the number of replacements in the protein-enriched scenario (scenario 2; protein-enriched) was much lower in comparison to scenarios 1 (protein rich) and 3 (combination). Therefore, scenario 2 seemed to be the most efficient scenario of this simulation model. This can be explained by the fact that protein-enriched foods often have a higher protein content compared to regular and natural foods since extra protein is added^(17)^. Hence, the effect of the scenarios is partly explained by the combination of foods within one scenario, but individual foods may have a considerable contribution to protein intake.

Despite a relevant increase in calculated protein intake, none of the scenarios achieved that 100 % of the participants had a mean protein intake of ≥ 1·0 g/kg BW/d. One explanation may be a very low protein intake in a small subgroup of participants. For this subgroup, the simulation model may increase the protein intake, but not sufficiently to meet a protein intake of 1·0 g/kg BW/d. Another explanation might be that some people consume little to no foods from the food groups that were included in the simulation model. In that case, no or few replacements were made.

Several intervention studies have been performed in the past that showed that protein intake increased remarkably when protein-enriched foods were offered, varying from 14 g to 42 g of extra protein/d^(18–22)^. This implies that shifting from regular foods to protein-enriched alternatives might be an effective way to increase protein intake among older adults. However, three of these studies included hospitalised older adults and older adults residing in a rehabilitation ward^(18–20)^. A study by Ziylan *et al.* ^(21)^ among community-dwelling older adults showed that protein intake increased by 14·6 g after replacing bread with protein-enriched bread and hot meals with protein-enriched readymade hot meals compared to the control group who received regular equivalents. A study by Borkent *et al.* ^(22)^ showed that protein intake increased by 13·6 g in participants who received readymade protein-rich hot meals and protein-rich dairy products compared to the control group who received standard readymade hot meals and drinks. Remarkably, participants in the control group decreased their protein intake compared to pre-study intake. Therefore, the authors concluded that switching from self-prepared meals to readymade meals could be a risk for a decreasing protein intake when readymade meals are not protein-enriched. In both studies^(18,22)^, changes were mainly made within the foods from the main meals (breakfast–lunch–dinner), but not in-between meals (snacks). Our simulation model included both foods consumed during main meals, as well as foods consumed in-between meals. This could explain the larger effect on protein intake in our study. However, since our simulation model is a theoretical framework, feasibility should be tested in practice.

The intervention studies that have been performed in community-dwelling older adults showed a smaller effect on protein intake compared to studies in hospitalised older adults. This may have several reasons. Research has shown that Dutch older adults tend to have low interest and willingness to purchase protein-enriched foods, and the price of protein-enriched foods is pointed out as a barrier to purchase those foods^(23,24)^. When offered (free of costs) at a hospital or rehabilitation centre, older adults might be more inclined to consume protein-enriched foods than when they have to purchase the foods at home. Additionally, older adults tend to be more sceptical towards protein-enriched foods and prefer consuming conventional foods that are naturally rich in protein^(23)^. However, our simulation model has shown that, to increase protein intake with foods that are naturally rich in protein, more replacements are needed compared to protein-enriched foods. All in all, more research is needed to evaluate the efficacy of intervention studies including protein-rich and protein-enriched foods and to identify all barriers and facilitators towards purchasing and consuming protein-rich and protein-enriched foods in the community setting.

The main goal of this study was to study the effects of a simulation model on protein intake. However, changing food patterns can alter the intake of energy and other macro- and micronutrients as well. Therefore, the impact of the replacements on intakes of energy and other macro- and micronutrients was also verified (Appendix I). Results show that energy increased moderately in all three scenarios, varying from 59 to 66 kcal/d. In practice, this could result in minor weight gain^(25)^. However, people may also compensate for the surplus of energy^(26)^. This, and the feasibility of the replacements within this simulation model should be tested through an intervention study. Contrarily, most macro- and micronutrients remained similar to the original scenario, with a few exceptions for the micronutrients copper, iodine, selenium, calcium and vitamin C. However, in the scenarios used in the simulation model, the majority of foods from one food group were replaced by just one specific alternative that may be rich or low in other macro- and micronutrients. This might have had a large effect on the total intake of that specific macro or micronutrient. In practice, it is unlikely that older adults will replace all foods in a food group with one specific alternative. All foods consumed in DNFCS with a protein content > P75 of the food group are shown in Appendix II. These findings can be used in dietary advice and/or in the development of new or existing foods.

### Strengths and limitations

To our knowledge, this study is one of the first studies to investigate possible strategies to improve protein intake in community-dwelling older adults through a simulation model. In this simulation model, the effect of hypothetical changes in the diet of Dutch community-dwelling older adults based on a priori decisions has been identified, keeping several practical issues in mind. A strength of this study is that the choice of the alternative foods was based on familiarity: foods that were already consumed by the target group and were high in protein content were chosen as alternative foods. Furthermore, the choice of the alternative foods was based on similarity: the alternative foods had to be similar to the foods they replaced (i.e. yoghurt and other desserts were replaced by quark, juices were replaced by yoghurt drink with a fruity flavour). Consequently, the alternative foods did not differ much from the products they replaced. Since the choice of the alternative foods was based on familiarity and similarity, the chance of adopting the changes into their current diet is enhanced. Another strength of this study was that foods were replaced within a food group by alternative foods in grams originally consumed instead of replacing foods with alternatives in portions. Thus, in practice, participants do not have to increase the portion size to increase protein intake. Recent studies have shown that enrichment of regular products does not affect satiety and has no impact on the consumption of other foods^(18,19,21)^. Consequently, it is assumed that participants eat similar amounts of food, even though food is protein-enriched. In this way, practical application is again more attainable. Lastly, a strength of this study was the estimation of habitual protein intake in the last step of the simulation model. In this way, within-person variation between the 2-d intake was removed, which led to a more accurate estimation of habitual intake of the original scenario and the protein-rich and protein-enriched scenarios.

Yet, this study also has some limitations. The dataset of the DNFCS-OA was the best data set available to include in this simulation model. However, the data originates from 2010 to 2012. Therefore, the data might not be representative of the current diet of Dutch older adults, as shown in previous research. Hulshof *et al.* (2003) found that intake of some foods and nutrients in Dutch subjects has changed significantly over 10 years^(27)^. On the other hand, it is known that older adults tend to keep a very stable food pattern over time^(28)^. The scenarios in this simulation model included replacements in six to nine food groups. Even though the actual intake of specific food groups might differ in the current population of Dutch older adults, the effects of the different scenarios might be similar, whereby the differences in intakes between the different food groups might compensate for the outcome. Furthermore, within the DNFCS-OA there is an overrepresentation of relatively healthy older adults^(7)^. Healthy older adults might have a higher protein intake compared to less healthy older adults^(29)^, while also the type of foods consumed may be different between healthy and less healthy older adults. Moreover, data of the DNFCS-OA are based on self-reported food intake. An error that often occurs in self-reported dietary intake is underreporting^(30)^. Therefore, actual protein intake might be higher than shown in the data of the DNFCS-OA.

## Conclusion

This study has revealed successful strategies to increase protein intake in the diet of community-dwelling older adults by replacing currently consumed foods low in protein with protein-rich and protein-enriched alternatives. Foods that have been used as a replacement of foods within a specific food group can be used in the formulation of dietary advice. However, other foods with a protein content above the P75 within that food group can also be used as a substitution. It is highly recommended to test the feasibility of the theoretical models used in this study in practice.

## Supplementary material

For supplementary material accompanying this paper visit https://doi.org/10.1017/S1368980021004134. click here to view supplementary material
