# Supplementary material for: The protein gap—increasing protein intake in the diet of community-dwelling older adults: a simulation study
Source: Public Health Nutr. 2021 Oct 4;25(2):248–56. doi: 10.1017/S1368980021004134 (PMC8883780; doi:10.1017/S1368980021004134)
Supplement: Supplementary file 1 [file S1368980021004134sup001.docx]

**Appendices**

| Appendix I: Mean micronutrient intakes and SD in the original scenario and in scenario 1, 2 and 3 * = Adequate intake; ** = Estimated Average Requirement; *** Maximum recommended intake | | | | | | | | | |
| --- | --- | --- | --- | --- | --- | --- | --- | --- | --- |
| Nutrient | Norm intakes^(32–37)^ | Average intake of 2 days | | | | | | |  |
|  |  | **Original scenario** | | **Scenario 1** | | **Scenario 2** | | **Scenario 3** | |
|  |  | **Mean** | **SD** | **Mean** | **SD** | **Mean** | **SD** | **Mean** | **SD** |
| Calcium (mg)* | 1200 | 966.6 | 324.3 | 1069.2 | 328.1 | 893.0 | 358.2 | 990.4 | 343.9 |
| Copper (mg)** | 0.7 | 1.1 | 0.4 | 1.5 | 0.5 | 1.0 | 0.5 | 1.3 | 0.5 |
| Iron (mg)** | 6 | 10.4 | 3.5 | 10.4 | 3.5 | 10.4 | 3.5 | 10.4 | 3.5 |
| Iodine (µg)* | 150 | 159.0 | 52.8 | 169.3 | 51.5 | 133.8 | 66.1 | 137.1 | 64.4 |
| Magnesium (mg)* | Men: 350  Women: 300 | 322.3 | 83.8 | 365.9 | 86.4 | 295.9 | 98.0 | 315.6 | 90.3 |
| Sodium (mg)*** | 2400 | 2329.4 | 804.7 | 2320.3 | 783.6 | 1996.7 | 923.0 | 2038. | 896.9 |
| Phosphorus (mg)* | 550 | 1406.9 | 357.7 | 1628.5 | 334.7 | 1297.9 | 429.3 | 1451.7 | 368.2 |
| Potassium (mg)* | 3500 | 3268.4 | 756.5 | 3689.6 | 801.1 | 3073.2 | 849.0 | 3423.7 | 808.2 |
| Selenium (µg)* | 70 | 44.7 | 17.6 | 76.0 | 36.9 | 42.5 | 18.6 | 71.6 | 34.7 |
| Zinc (mg)** | Men: 6.4  Women: 5.7 | 10.1 | 3.2 | 11.8 | 3.0 | 9.5 | 3.5 | 10.7 | 3.2 |
| Retinol activity equivalents (µg)* | Men: 615  Women: 525 | 1026.1 | 1592.8 | 927.4 | 1590.9 | 996.9 | 1595.0 | 927.9 | 1591.1 |
| Folate equivalents (µg)** | 200 | 330.8 | 143.6 | 336.1 | 141.0 | 314.1 | 148.5 | 311.0 | 146.9 |
| Vitamin B1 (mg)** | 0.85 | 1.1 | 0.5 | 1.2 | 0.44 | 1.0 | 0.5 | 1.1 | 0.5 |
| Vitamin B2 (mg)** | Men: 1.5  Women: 1.1 | 1.4 | 0.5 | 1.6 | 0.5 | 1.3 | 0.5 | 1.4 | 0.5 |
| Vitamin B6 (mg)** | Men: 1.3  Women: 1.1 | 1.8 | 0.7 | 1.8 | 0.6 | 1.7 | 0.7 | 1.8 | 0.6 |
| Vitamin C (mg)** | Men: 60  Women 50 | 104.4 | 56.6 | 83.6 | 51.2 | 101.3 | 56.4 | 81.5 | 52.0 |
| Vitamin D (µg)* | 61 – 70y: 10  >70y: 15 | 4.1 | 2.8 | 4.0 | 2.8 | 4.0 | 2.9 | 4.1 | 2.8 |
| Vitamin E (mg)* | Men: 13  Women: 11 | 12.9 | 5.7 | 12.1 | 5.7 | 12.3 | 5.7 | 12.2 | 5.7 |

| Appendix II: Foods with a protein content > p75, per food group with corresponding NEVO code | | |
| --- | --- | --- |
| **Food group** | **Foods with protein intake >p75** | **NEVO code** |
| **Savoury snacks** | Almonds blanched unsalted/unsalted  Cashew nuts unsalted/salted  Peanuts unsalted/salted/dry roasted  Nuts mixed unsalted/salted  Sesame seeds  Linseeds  Pistachio nuts salted  Pine nuts  Wrap shoarma roll prep wo fat  Pumpin seeds | 198/2887  199/2886  204/876/2048  207/1935  838  867  1896  2176  2550  2806 |
| **Breakfast cereals** | Oatmeal  Breakfast cereal Brinta  Breakfast cereal All-Bran Plus Kellog’s  Breakfast product Special K Original  Breakfast product Albona 7-cereals-energy | 213  225  591  2005  2361 |
| **Milk and milk products** | Yoghurt low fat  Pudding vanilla  Mousse chocolate  Pudding chipolata  Yoghurt full fat with fruit  Yoghurt Bulgarian low fat  Fromage frais half fat w fruit  Blancmange vanilla w strawberry sauce  Yoghurt half fat  Fromage frais low fat w fruit w sw  Fromage frais yoghurt w fruit  Tiramisu  Yoghurt Greek full fat  Pudding airy average  Porridge milk w flour Lammetjespap  Yoghurt & custard Campina  Yoghurt 0% fat w fruit Activia  Porridge oatmeal w semi-skimmed milk  Milk raw  Milk chocolate-flavoured full fat  Milk chocolate-flavoured low fat  Milk whole  Milk full fat condensed w sugar tinned  Milk semi-skimmed  Buttermilk  Milk skimmed  Milk skimmed dried  Milk whole dried  Buttermilk with fruit  Yoghurt drink  Milk chocolate-flavoured semi-skimmed  Milk semi-skimmed enriched w calcium  Milk chocolate-flavoured Chocomel light  Milk goats- full fat  Yoghurt drink with sweeteners  Coconut milk  Drinking chocolate w s-sk milk  Dairy drink Campina fruitmilk  Drinking chocolate w s-sk milk +Nesquik  Milkdrink skimmed milk Becel pro-activ  Hot chocolate from vending machine  Coffee iced  Dairy drink Milk&Fruit mango  Milk chocolate-flavoured w sw Optimel | 301  736  767  786  863  916  917  940  1502  2246  2247  2371  2503  2520  2521  2536  2655  3050  270  272  273  279  281  286  289  294  295  296  479  657  1464  1719  1970  2240  2254  2290  2495  2496  2500  2725  2760  2835  2917  3004 |
| **Soups** | Soup clear with meat  Soup clear with meat and noodles  Soup clear with meat and vegetables  Soup clear with meat vegetables and noodles  Soup main course with legumes and meat  Stock powder low sodium | 758  760  761  762  766  1883 |
| **Vegetables and legumes** | Kale curly boiled  Mushrooms chanterelle boiled  Mushrooms boiled  Lettuce head boiled  Swiss chard leaf boiled  Spinach raw  Bean sprouts raw  Bean sprouts boiled  Peas garden medium fine tinned  Peas garden super fine tinned  Peas and carrots tinned  Spinach tinned  Tomato puree concentrated tinned  Beans broad tinned  Peas marrowfat legumes tinned  Beans baked in tomato sauce tinned  Swiss chard leaf raw  Spinach creamed frozen boiled  Beans brown tinned  Garlic fresh  Broccoli boiled  Peas frozen boiled  Beans broad boiled  Peas fresh boiled  Beans white/brown boiled  Lentils boiled  Peas green boiled  Dandelion leaves raw  Peas chick boiled  Peas and carrots frozen unprepared  Vegetable mixed Mexico frozen unprepared  Beans runner frozen unprepared  Beans broad frozen unprepared  Onions deep-fried sachet  Tomatoes dried in oil tin/glass  Tomato sun-dried  Rocket raw  Sweetcorn tinned  Beans white tinned | 16  18  20  47  48  51  58  59  134  135  136  140  141  142  196  197  563  651  660  830  920  953  962  963  968  970  972  1087  1095  1139  1141  1143  1148  1484  2377  2378  2736  2900  3049 |
| **Cheese** | Cheese Swiss dried  Cheese Edam 40+  Cheese 20+ Leidse w cumin/Fries clove  Cheese Parmesan  Cheese Gruyere  Cheese Emmentaler  Cheese Cheddar  Cheese Amsterdam 48+  Cheese raw milk 48+  Cheese sodium reduced 48+  Cheese 30+  Cheese 20+  Cheese Leerdammer/Maasdammer 45+  Cheese 40+ Leiden w cumin/Fries clove  Cheese 45+  Cheese 30+ low salt | 304  511  514  718  722  724  725  883  1112  1113  1382  1723  1725  1726  1809  2824 |
| **Pastry and biscuits** | Biscuit fortified Liga Tweede Stap  Almond filled pastry  Biscuit sweet  Cake wo butter  Cake sponge Dutch Eierkoek  Biscuit sponge fingers  Biscuit brown/wholemeal  Doughnut Dutch style  Biscuit oatmeal  Biscuit spiced Speculaas w almond paste  Biscuit chocolate  Biscuits sugar free  Biscuit fortified Liga Milkbreak  Cake made with butter  Cake Dutch spices ontbijtkoek w nuts  Wafer galette  Wafer w milk & hazelnts Knoppers  Cake with nuts  Biscuit fortified LU Time Out  Cake with “bitterkoekjes”  Biscuit Bridge ommetjes  Sponge cake wholemeal | 234  250  252  253  254  260  263  474  837  855  1471  1477  1965  1969  2397  2425  2428  2432  2556  2571  2719  2933 |
| **Sugar, sweets, sweet spreads and sweet sauces** | Cocoa powder  Chocolate milk  Candybar Snickers  Cocoa product powder Ovomaltine  M&M’s chocolate with peanuts  Chocolate bar milk with nuts  Almond paste with egg  Chocolate plain with nuts  Chocolate milk w puffed rice  Cocoa product sweetened Nesquik Hot Choc | 430  431  528  595  621  717  790  2375  2376  2415 |
| **Bread** | Bread wholemeal average  Bread wholemeal w pumpkin seeds  Break linseed  Bread multigrain average w seeds  Bread wholemeal w nuts  Bread wholemeal w sunflower seeds  Bread wholemeal w seeds  Bread brown w seeds  Bread corn w sunflower seeds  Roll brown hard  Roll brown soft  Rolle wholemeal soft  Roll multigrain hard  Roll multigrain soft  Bread brown w pumpkin seeds  Croissant chocolate-  Almond paste filled tarts w butter  Croissant prepared w butter  Croissant average  Bread brioche | 246  2348  2349  2350  2354  2357  2703  2704  2707  2796  2797  2798  2799  2800  2821  2400  2761  2801  2818  2876 |
| **Non-alcoholic drinks** | Juice tomato/vegetable Appelsientje  Juice tomato Appelsientje Zontomaat | 1933  1934 |
